# Confronting the inevitable: Harnessing technology to contain systemic scientific fraud

**DOI:** 10.1073/pnas.2521606122

**Published:** 2025-10-27

**Authors:** Philipp Singer

**Affiliations:** ^a^Roche Diagnostics International AG, Knowledge Strategy & Language Operations, Rotkreuz 6343, Switzerland

The crisis outlined by Richardson et al. (1) makes one thing painfully clear: Fraud is no longer a series of isolated ethical lapses but a business model that exploits vulnerable points in the research economy. Because publication and citation metrics remain the coin of academic success, the literature is tilted toward positive results; this “upright” bias magnifies false-positive findings and slows truly novel discoveries that would emerge from negative or null studies. Fighting that statistical gravity is hard enough—fighting it while sophisticated paper mills churn out fabricated data is harder still.

Humans have always been tempted to cut ethical corners for personal gain; today, however, that temptation has been productized. Commercial “paper mills” and increasingly capable text-generating AIs can now mass-produce manuscripts that look passably real. The marginal cost of fraud is dropping toward zero, while the cost of traditional peer review remains stubbornly high. Fraud, in short, scales.

Yet, the same machines that automate deceit can help automate its detection. Richardson’s team showed that network-level forensics—cross-checking implausible author–editor ties, anomalous citation patterns, and image similarities—can expose paper-mill products at scale.

Those algorithms can and should be baked into editorial pipelines, flagging suspect manuscripts before they reach PubMed or major indexes. Because false positives carry reputational risk, journals could publish machine-generated “fraud likelihood scores” alongside reviewer reports, creating transparent incentives to clean house.

Market forces also matter. A journal that cannot guarantee authenticity will shed credibility and, eventually, impact factor. Funders face a parallel risk: Grants built on corrupted evidence waste money and erode public trust. Both groups therefore have leverage to demand stronger data-integrity guarantees. Concretely:

- Mandatory raw-data escrow—Principal investigators should deposit immutable, timestamped raw data in certified repositories before first submission. Peer reviewers and, later, independent auditors must have blind access.
- Conditional funding—Agencies such as the NIH or U.S. Food and Drug Administration (FDA) should withhold payments (or enforce claw-backs) unless raw data remain available for a fixed period and survive random spot audits.
- Automated integrity certificates—Just as HTTPS certificates vouch for a website, journals could issue machine-readable attestations that a paper has passed image-duplication and text-similarity screens, signed with a public key.

Will this eliminate fraud? No, human nature is not patchable. But complex systems often adapt via negative feedback. Once fraud reaches an epidemiological tipping point—as many fear it soon will—the reputational and financial costs imposed by funders, journals, and even citation-analysis platforms will grow too large for institutions to ignore. The scientific immune system may be slow, but history shows it is ruthless once activated: Retractions, de-indexing, and grant freezes eventually isolate the offenders, allowing credible actors to reclaim the commons.

The task, then, is to accelerate that immune response. By pairing transparent incentives with automated policing—fighting systemic fraud with the very algorithms that enable it—we can shorten the period during which fabricated results distort the record, misdirect resources, and delay lifesaving treatments. Fraud may be inevitable; its dominance need not be.
